# Modeling the Synergistic Impact of Yttrium 90 Radioembolization and Immune Checkpoint Inhibitors on Hepatocellular Carcinoma

**DOI:** 10.3390/bioengineering11020106

**Published:** 2024-01-23

**Authors:** Minah Kang, Yerim Shin, Yeseul Kim, Sangseok Ha, Wonmo Sung

**Affiliations:** 1 Department of Biomedicine and Health Sciences, College of Medicine, The Catholic University of Korea, Seoul 06591, Republic of Korea; minah.kang@catholic.ac.kr (M.K.); angela3shin@catholic.ac.kr (Y.S.); yeseulkim@catholic.ac.kr (Y.K.); sangseok@catholic.ac.kr (S.H.); 2 Department of Biomedical Engineering, College of Medicine, The Catholic University of Korea, Seoul 06591, Republic of Korea

**Keywords:** immune checkpoint inhibitor, radioembolization, mathematical modeling

## Abstract

The impact of yttrium 90 radioembolization (Y90-RE) in combination with immune checkpoint inhibitors (ICIs) has recently gained attention. However, it is unclear how sequencing and dosage affect therapeutic efficacy. The purpose of this study was to develop a mathematical model to simulate the synergistic effects of Y90-RE and ICI combination therapy and find the optimal treatment sequences and dosages. We generated a hypothetical patient cohort and conducted simulations to apply different treatments to the same patient. The compartment of models is described with ordinary differential equations (ODEs), which represent targeted tumors, non-targeted tumors, and lymphocytes. We considered Y90-RE as a local treatment and ICIs as a systemic treatment. The model simulations show that Y90-RE and ICIs administered simultaneously yield greater benefits than subsequent sequential therapy. In addition, applying Y90-RE before ICIs has more benefits than applying ICIs before Y90-RE. Moreover, we also observed that the median PFS increased up to 31~36 months, and the DM rates at 3 years decreased up to 36~48% as the dosage of the two drugs increased (*p* < 0.05). The proposed model predicts a significant benefit of Y90-RE with ICIs from the results of the reduced irradiated tumor burden and the associated immune activation and suppression. Our model is expected to help optimize complex strategies and predict the efficacy of clinical trials for HCC patients.

## 1. Introduction

Hepatocellular carcinoma (HCC), which is the most common primary liver cancer, is a leading cause of cancer-associated death worldwide [1,2]. The traditional treatments for early-stage tumors consist of surgical resection, liver transplantation, and locoregional treatments, and intermediate-stage tumors are treated with transarterial chemoembolization (TACE) and tyrosine kinase inhibitors (TKIs) such as sorafenib and lenvatinib [3,4,5]. Despite continuous advancements in treatment modalities and research, the median overall survival (OS) for the intermediate stage of HCC is only 20~30 months, and that for advanced-stage HCC is 10~19 months [6,7]. The majority of patients with HCC have unresectable disease at early diagnosis and lose the opportunity for curative treatment [8,9]. Thus, a novel approach to the treatment of HCC is needed to improve clinical outcomes.

Cancer immunotherapy has emerged as a promising treatment for cancer [10]. Tumors evade the immunological surveillance system in many ways and proliferate in the tumor microenvironment while inhibiting the activation of immune suppression [11]. The identification of the immune checkpoint mechanism has played a significant role in the development of immune checkpoint inhibitors (ICIs), including cytotoxic T lymphocyte-associated antigen 4 (CTL-4), programmed cell death 1 (PD-1), and programmed cell death ligand 1 (PD-L1) inhibitors, which provide new opportunities to treat advanced HCC [12,13,14,15,16]. Atezolizumab (anti-PD-L1) and Bevacizumab (anti-vascular endothelial growth factor) significantly improved progression-free survival (PFS) outcomes (6.8 vs. 4.3 months) and OS (19.2 vs. 13.4 months) against sorafenib [12,13]. The phase III HIMALAYA trial demonstrated that a combination of tremelimumab (anti-CTLA-4) and durvalumab (anti-PD-L1) showed better OS (16.42 vs. 13.77 months) versus sorafenib. Additionally, durvalumab monotherapy was not inferior to sorafenib for OS [14].

The yttrium 90-radioembolization (Y90-RE) can stimulate the release of tumor antigens, which can activate T cells and enhance anti-tumor immunity [17], induce immunogenic cell death resulting in tumor regression in distant non-irradiated areas [18,19], and change the tumor microenvironment [20]. As Y90-RE has been shown to activate the immune system, further investigations are needed to evaluate the effectiveness and safety of Y90-RE and ICI combination treatment for HCC patients [21,22,23,24,25,26,27,28].

Despite the increasing number of clinical trials [21,22,23,24,25,26,27,28], there remains an unresolved concern with the combination of Y90-RE and ICIs regimens, as preclinical data suggests that strategic clinical studies on the type of agents, dosage, and sequence are required [29,30]. Mathematical modeling can be used in in silico virtual clinical trials to assist in the design of actual treatment because combining multiple modalities in clinical trials is a costly, time-consuming, and labor-intensive form of research procedure. To our knowledge, there is no mathematical model that can simulate the therapeutic effects of different sequences and dosages of Y90-RE and ICIs on the tumor-immune systems. In this study, we developed a mathematical model to quantify and predict the synergistic effects of Y90-RE and ICI combination therapy and investigated their efficacy to find optimal treatment sequences and dosages.

## 2. Materials and Methods

### 2.1. Mathematical Model Overview

**Figure 1:**
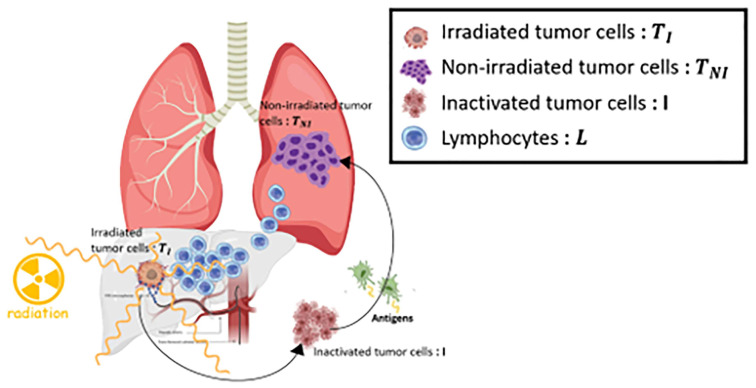
A schematic representation of the model. The model has four compartments: targeted tumor cells ($T_{I}$), inactivated tumor cells (I), non-targeted tumor cells ($T_{NI}$), and circulating lymphocytes (L). The targeted tumor cell and circulating lymphocytes are exposed to radiation emitted from the decay of yttrium 90, but not non-targeted tumor cells. Both targeted and non-targeted tumor cells are cytotoxic according to immune response. The immune response can be upregulated by the administration of Programmed Death-Ligand 1 (PD-L1) immune checkpoint inhibitors (durvalumab).

We used a model taken from a previous study that combines ICI treatment with external radiation therapy [31] to simulate the ICI and Y90 RE combination therapy. The model is made up of four compartments: (1) targeted tumor cells $T_{I}$, (2) non-targeted tumor cells $T_{NI}$, (3) inactivated tumor cells that release antigens *I*, and (4) circulating lymphocytes, *L*, which are described with ordinary differential equations (ODEs) that were modified from the predator–prey model [32,33,34]. In this study, the impacts of tumor cell death resulting from the immune system, tumor cell growth exponential, tumor cells killed by linear–quadratic radiation, and circulating immune cells are taken into account. While immune-induced cell death affects both tumor compartments, radiation-induced cell death impacts only the targeted one, resulting in inactivated tumor cells (I). In this model, Y90-RE inactivates cells proportionally, and the number of tumor-directed effector lymphocytes (L) grows with tumor antigen presentation; otherwise, the number of these cells decays steadily. Figure 1 shows a summary of the biological assumptions and a schematic overview of the model. The complete set of model equations is provided in (1)–(5) (detailed in Supplementary Section S1). Our model separates the targeted and non-targeted tumor compartments. Both local and systemic treatments can affect the number of targeted tumor cells, but only systemic treatments can affect the number of non-targeted tumor cells. In this study, we assumed that Y90-RE was treated as a local treatment and ICIs as a systemic treatment. Therefore, the non-targeted tumor is usually metastasized and outside the Y90-RE area. The compartments are separated to simulate their differential dynamics resulting from the several therapies impacting them, as well as because their sizes can be measured and approximated for individual patients.

(1) $$\frac{dT_{I}}{dt}=\overset{Tumor\;growth}{\overbrace{aT_{I}}}\;\;\overset{immune-induced\;death}{\overbrace{-\omega_{1}^{\prime }\frac{T_{I}}{g+T_{I}+M}L}}\;\;\;\;\overset{radiation\;kill\;from\;Yttrium-90}{\overbrace{-\delta^{*}\left(t_{R}\right)\left(1-e^{-\alpha_{T}D_{T}{-\beta }_{T}{D^{2}}_{T}}\right)\;T_{I}}}$$

(2) $$\frac{dT_{NI}}{dt}=\overset{Tumor\;growth}{\overbrace{aT_{NI}}}\;\;\overset{immune-induced\;death}{\overbrace{-\omega_{1}^{\prime }\frac{T_{NI}}{g+T_{I}+T_{NI}}\;L}}$$

(3) $$\frac{dI}{dt}=\overset{radiation\;kill\;from\;Yttrium-90}{\overbrace{-\delta^{*}\left(t_{R}\right)\left(1-e^{-\alpha_{T}D_{T}{-\beta }_{T}{D^{2}}_{T}}\right)\;T_{I}}}\;\;\;\;\overset{decay}{\overbrace{-rI}}$$

(4) $$\frac{dL}{dt}=\overset{recruitment}{\overbrace{\omega_{2}\frac{T_{I}+T_{NI}}{g+T_{I}+T_{NI}}\;L+\omega_{3}\frac{I}{g+I}\;L}}\;\;\;\overset{supply}{\overbrace{+s}}\;\;\overset{decay}{\overbrace{-fL}}\;\;\overset{radiation\;kill\;from\;Yttrium-90}{\overbrace{-\sum_{i}\delta^{*}(t_{R})(1-e^{{-\alpha }_{L}D_{L_{i}}})L_{i}}}$$

(5) $$\omega_{1}^{\prime }(t)=\overset{immune\;checkpoint\;inhibitors-induced\;death}{\overbrace{\omega_{1}(1+\delta_{durva}\cdot C_{max}e^{\frac{-\ln\left(2\right)t}{t_{(\omega )1/2}}})}}$$

Radiation cell death is represented discretely by instantaneous change at distinct timepoints with the Dirac delta function $\delta^{*}$($T_{R})$, instead of solving ordinary differential Equations (1)–(5). A linear quadratic (LQ) model is utilized to explain cell survival for the tumor cells (6) and (7), and a linear survival model is employed to describe the killing of immune cells (8).

We add a new formalism (9) shown in red to describe the exponential radioactive decay of Y90-RE while the radiation is delivered to the targeted tumor cells [35,36] by extending the previously developed ICIs-RT model [31]. <*E*> is the average energy emitted per nuclear transition, m is the mass of tissue that absorbs the radiation, and k is a constant that produces the dose rate in the desired units. *A*_0_ is the activity in the target mass of interest and T(Y90−RE)12 is the half-life of the radioactive source. The total number of disintegrations, commonly known as cumulated activity, is equal to *A*_0_ × *T*1/2/ln (2).

In all areas of interest, activity is considered to be distributed equally. It is also assumed that there is no bremsstrahlung creation in beta particle decay and that the entire amount of decay energy is absorbed by the mass. The effective half-life is the same as the radioactive half-life, as there is no need to remove the radioactive source from the patient.

(6) $$T_{n+1}=T_{n}\cdot e^{-\alpha_{T}D_{T}-\beta_{T}{D_{T}}^{2}}$$

(7) $$I_{n+1}=I_{n}+T_{n}\cdot (1-e^{-\alpha_{T}D_{T}-\beta_{T}{D_{T}}^{2}})$$

(8) $$L_{n+1}=\sum_{i}{L_{n}}_{i}\cdot e^{-\alpha_{L}D_{L_{i}}}$$

(9) $$D_{T}=q\cdot \frac{k\;\cdot <E>\cdot A_{0}}{m}\int_{0}^{\infty }e^{-\frac{ln2}{T(Y90)1/2}}$$

All parameters taken from the previously developed ICIs-RT model [31] and the newly developed term ($D_{T}$) in this study are listed in Table 1, and the newly added parameters are highlighted in gray shading.

### 2.2. Virtual Patient Cohort

The same cohort of virtual population from the previous study [46] was implemented in this study to simulate the combination therapy of Y90-RE and ICIs. All of the patient populations have an inherent set of distributions for tumor volume, circulating lymphocytes, and radiosensitivity representing HCC patients, which reflect interindividual variation and heterogeneity in treatment outcome. The Python programming language (Python Software Foundation, Version 3.9, Wilmington, DE, USA) was used to implement random sampling generation from the normal distribution and to simulate a mathematical model with the virtual patients. Targeted tumor density was assumed to be 10^9^ times the number of tumor cells per cubic cm. The number of non-targeted tumor cells was estimated to be 0.1% ($1.07\times 10^{8})$ of the targeted tumor volume. We calculated the average baseline number of circulating lymphocytes (5.61 × $10^{9}$) by multiplying the average lymphocyte counts (1122 ± 469 per ${mm}^{3}$) with the total body volume (5 L). The distributions of initial tumor size ($T_{0}$), circulating lymphocytes ($L_{0}$), and tumor radiation sensitivity (*αT*) are illustrated in Supplementary Figure S1.

### 2.3. Model Fitting to Yttrium 90 Radioembolization Therapy Response Data

We validated that the new Y90-RE single model works correctly. The Y90-RE parameters were fitted independently for clinical trial data in HCC patients receiving Y90-RE treatment [47,48].

Based on the Response Evaluation Criteria in Solid Tumors (RECIST), patient response to treatments can be defined into four categories: complete response (CR, removal of all target lesions), partial response (PR, at least 30% decrease in the sum of longest diameters of target lesions in comparison to the baseline value), progressive disease (PD, at least 20% rise in the sum of longest diameters of target lesions in comparison to the baseline value), and stable disease (SD, when none of the above criteria fits the tumor response), as in two clinical trials [47,48]. To determine the probability distributions of q, which represents the constant treatment effectiveness of Y90-RE, we generated a cohort of 10,000 patients with a fixed range of q between 0 and 1. Then, we assessed the RECIST 1.1 responses by measuring the size of the tumor at 6 months after the first Y90-RE treatments. We found the distinct q distribution that corresponds to the clinical response observed in the Y90-RE trials [47,48], as determined by the estimated RECIST 1.1 responses: CR: 79%, PR: 20%, SD: 0%, and PD: 0% (Supplementary Figure S2). The estimated mean and standard deviation of the q distribution are 0.12 and 0.04 (Supplementary Figure S3), which are for all Y90-RE and ICI combination simulations.

### 2.4. Virtual Clinical Trial and Outcome

**Figure 2:**
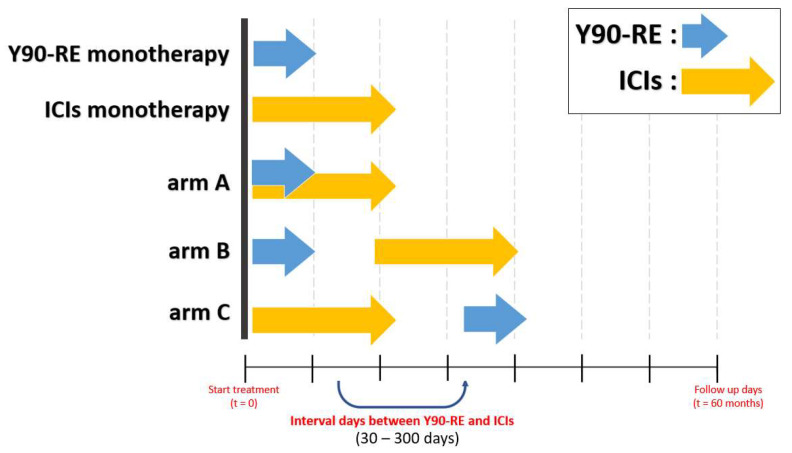
A schematic virtual trial schedule of the model with five different treatment groups. The blue arrows indicate the administrating timing of Y90-RE, and the yellow arrows indicate the administrating period of ICIs. Arms A, B, and C are shown according to the order in which Y90-RE and ICIs were administered, respectively. The administration interval days between the two drugs were investigated from 30 days to 300 days.

The virtual clinical trials consisted of five treatment groups: Y90-RE monotherapy, ICI monotherapy, and three groups for the combination regimens of Y90-RE and ICIs corresponding to the three arms (A, B, and C). Arm A involved the simultaneous administration of Y90-RE and ICIs. In arm B, before the administration of ICIs, Y90-RE was given. In arm C, before the administration of Y90-RE, ICIs were given. For Y90-RE, the fixed total radiation absorbed dose to the target volume at 300 Gy to 500 Gy was given. For ICIs, 0.08 to 0.16 of durvalumab (*δ_durva_*) was given every 2 weeks for 2 years. We investigated the effect on treatment outcome according to the timing of the Y90-RE and ICI administration interval days. The administration interval days between Y90-RE and ICIs were evaluated from 30 days to 300 days. All five treatments are depicted schematically in Figure 2.

The primary endpoints were progression-free survival (PFS) for targeted tumors and the cumulative distant metastasis (DM) rate for non-targeted tumors. PFS and DM rates were estimated using the Kaplan–Meier method. PFS was measured from the date of targeted tumor volume to progression or death, and DM rates were measured when the non-targeted tumor volume was greater than 0.1 cc, which can be observed with current diagnostic technology. A total of 4064 patients (40%) with the non-targeted tumor had over 0.1 cc. Both endpoints were evaluated using log-rank analysis for comparison, with a *p* value < 0.05 considered statistically significant.

## 3. Results

We conducted computer simulations on virtual patients with five different treatment regimens to predict the efficacy of the therapy modality. The virtual patients were simulated using each treatment protocol outlined in the material and method. All simulations were performed with Python version 3.9.

The combined regimens of arm A had longer PFS (*p* < 0.05) than those groups of Y90-RE monotherapy and ICI monotherapy for the targeted tumors. The median PFS from arm A was 39.3 months (95% CI, 37.2–39.2 mo), with Y90-RE monotherapy having a median of 16.2 months (95% CI, 16.3–17 mo) and ICI monotherapy having a median of 6.2 months (95% CI, 5.8–6.6 mo). Likewise, arm A had significantly lower cumulative DM rates at 3 years (*p* < 0.05) than those groups of Y90-RE monotherapy and ICI monotherapy for the non-targeted tumors. The DM rates at 3 years from arm A were 79.7% (95% CI, 77.2–82.2%), and the DM rates at 3 years from Y90-RE monotherapy and ICI monotherapy were not available (NA) and 99.9% (95% CI, 98.7%–NA), respectively (Supplementary Figure S4).

**Figure 3:**
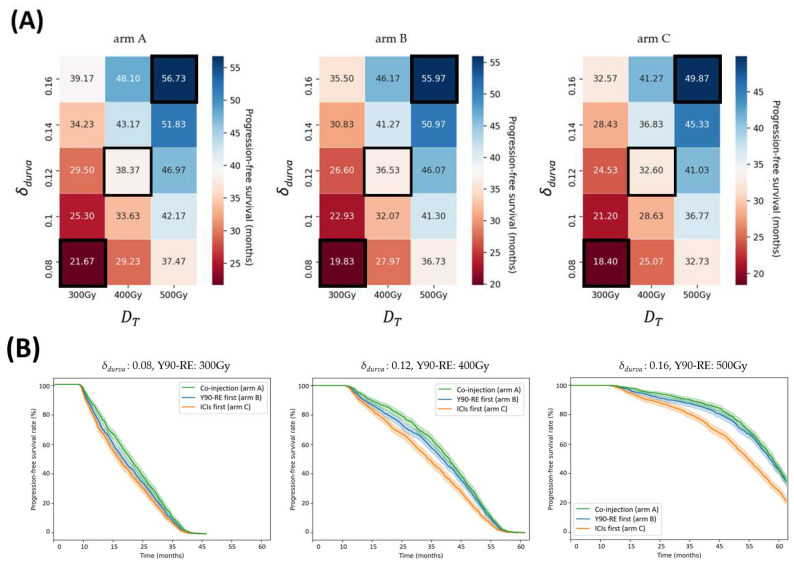
(**A**) The heatmap of median progression-free survival (PFS) for different dosages of Y90-RE and ICIs in the three arms (A, B, and C). The radiation intensity varies in the range of 300–500 Gy, and the PD-L1 (*δ_durva_*) varies in the range of 0.08–0.16. The color columns represent the median PFS. The black frames represent the minimum, median, and maximum points of the drugs, respectively, and are shown in graph (**B**). (**B**) Kaplan–Meier graph represents PFS for arm A (green), arm B (blue), and arm C (orange). Abbreviation: Y90-RE = yttrium 90 radioembolization; ICIs = immune checkpoint inhibitors.

**Figure 4:**
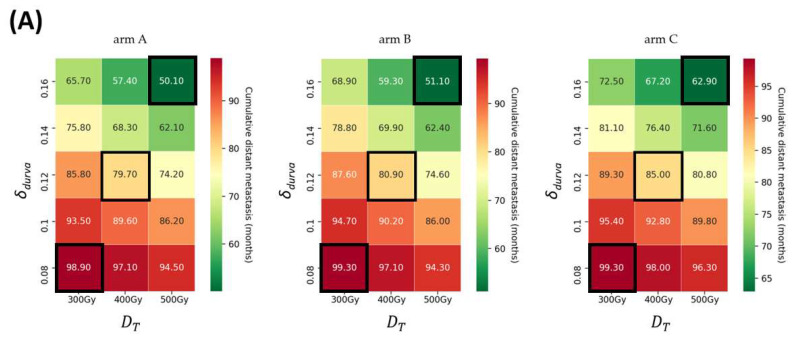

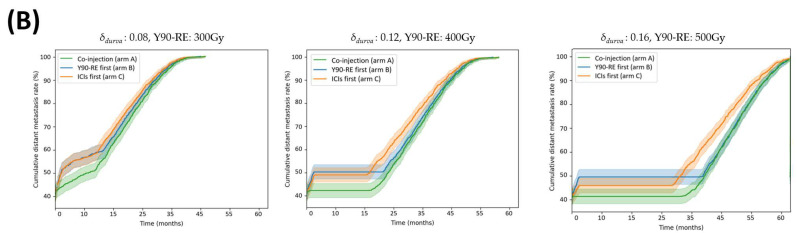
(**A**) The heatmap of the cumulative distant metastasis (DM) at 3 years for different dosages of Y90-RE and ICIs for three arms (A, B, and C) for non-targeted tumors. Radiation intensity varies in the range of 300–500 Gy, and the PD-L1 (*δ_durva_*) varies in the range of 0.08–0.16. The color columns represent the DM rates at 3 years. The black frames represent the minimum, median, and maximum points of the drugs, respectively, and are shown in graph (**B**). (**B**) The cumulative DM curve for arm A (green), arm B (blue), and arm C (orange). Abbreviation: Y90-RE = yttrium 90 radioembolization; ICIs = immune checkpoint inhibitors.

The present study reveals a robust correlation between dosage for targeted and non-targeted tumors. Figure 3A and Figure 4A show the efficacy heatmaps for the three arms (A, B, and C): the radiation intensity varies in the range of 300–500 Gy, and the PD-L1 (*δ_durva_*) varies in the range of 0.08–0.16. The color column in Figure 3A represents the median PFS, and the color column in Figure 4A shows the 3-year DM rates. According to the treatment schedules for arm B and arm C, the administration of Y90-RE and ICIs was carried out with 60 interval days for each.

For the patients who have targeted tumors, the simulation results showed that arm A yields the best efficacy (*p* < 0.05). Specifically, as PD-L1 or the dosage of Y90-RE increases, the median PFS increases up to 31~36 months. Arms A, B, and C demonstrated promising efficacy in that order. The efficacy maps for arm A, arm B, and arm C in Figure 3A show that the order in which these two treatments are given does make a significant difference to the outcome.

For 0.08 of *δ_durva_* and 300 Gy of Y90-RE, the median PFS from arms A, B, and C were 21.6 months (95% CI, 20.8–22.6 mo), 19.8 months (95% CI, 18.8–20.6 mo), and 18.4 months (95% CI, 17.6–19.2 mo), respectively. For 0.12 of *δ_durva_* and 400 Gy of Y90-RE, the median PFS from arms A, B, and C were 38.4 months (95% CI, 37.2–39.2 mo), 36.5 months (95% CI, 35.3–37.5 mo), and 32.6 months (95% CI, 31.4–33.8 mo), respectively. For 0.16 of *δ_durva_* and 500 Gy of Y90-RE, the median PFS from arms A, B, and C were 56.7 months (95% CI, 55–57.7 mo), 55.9 months (95% CI, 54.2–57.1 mo), and 46.5 months (95% CI, 48.2–51.1 mo), respectively.

In arm A, the 1-, 3-, and 5-year PFS rates from 0.08 of *δ_durva_* and 300 Gy of Y90-RE were 83.2% (95% CI, 80.7–85.3%), 3.2% (95% CI, 2.2–4.4%), and NA. The 1-, 3-, and 5-year PFS rates from 0.12 of *δ_durva_* and 400 Gy of Y90-RE were 96.4% (95% CI, 95–97.3%), 54.9% (95% CI, 51.8–58%), and NA. The 1-, 3-, and 5-year PFS rates from 0.16 of *δ_durva_* and 500 Gy of Y90-RE were 99.9% (95% CI, 99.2–99.8%), 88.5% (95% CI, 86.2–90.2%), and 33.8% (95% CI, 30.9–36.8%).

In arm B, the 1-, 3-, and 5-year PFS rates from 0.08 of *δ_durva_* and 300 Gy of Y90-RE were 78.2% (95% CI, 75.6–80.7%), 2.2% (95% CI, 1.4–3.2%), and NA. The 1-, 3-, and 5-year PFS rates from 0.12 of *δ_durva_* and 400 Gy of Y90-RE were 94.7% (95% CI, 93.2–96%), 49.9% (95% CI, 46.9–53.1%), and NA. The 1-, 3-, and 5-year PFS rates from 0.16 of *δ_durva_* and 500 Gy of Y90-RE were 99.8% (95% CI, 99.2–99.8%), 85.8% (95% CI, 83.4–87.8%), and 34.1% (95% CI, 31.1–37.0%).

In arm C, the 1-, 3-, and 5-year PFS rates from 0.08 of *δ_durva_* and 300 Gy of Y90-RE were 74.8% (95% CI, 71.9–77.3%), 1.6% (95% CI, 0.9–2.5%), and NA. The 1-, 3-, and 5-year PFS rates from 0.12 of *δ_durva_* and 400 Gy of Y90-RE were 92.6% (95% CI, 90.7–94%), 41.2% (95% CI, 38.1–44.1%), and NA. The 1-, 3-, and 5-year PFS rates from 0.16 of *δ_durva_* and 500 Gy of Y90-RE were 99.7% (95% CI, 99.2–99.9%), 76.9% (95% CI, 74.1–79.3%), and 21.1% (95% CI, 18.6–23.6%).

Likewise, for the patients who have non-targeted tumors, arm A had lower 3-year DM rates than arms B and C (*p* < 0.05). In the sequential treatment, arm B had lower 3-year DM rates than arm C (Figure 4A). With an increase in PD-L1 or dosage of Y90-RE, the cumulative DM rates at 3 years decreased by 36.4 to 48.8%.

In arm A, the 1-, 3-, and 5-year DM rates from 0.08 of *δ_durva_* and 300 Gy of Y90-RE were 53% (95% CI, 50–56.2%), 98.9% (95% CI, 98.1–99.95%), and NA. The 1-, 3-, and 5-year DM rates from 0.12 of *δ_durva_* and 400 Gy of Y90-RE were 42.3% (95% CI, 39.4–45.5%), 79.8% (95% CI, 77.3–82.3%), and NA. The 1-, 3-, and 5-year DM rates from 0.16 of *δ_durva_* and 500 Gy of Y90-RE were 41.7% (95% CI, 38.8–44.9%), 50.2% (95% CI, 47.2–53.4%), and NA.

In arm B, the 1-, 3-, and 5-year DM rates from 0.08 of *δ_durva_* and 300 Gy of Y90-RE were 59.0% (95% CI, 55.9–62.0%), 99.3% (95% CI, 98.7–99.7%), and NA. The 1-, 3-, and 5-year DM rates from 0.12 of *δ_durva_* and 400 Gy of Y90-RE were 50.4% (95% CI, 47.4–53.6%), 80.9% (95% CI, 78.6–83.4%), and NA. The 1-, 3-, and 5-year DM rates from 0.16 of *δ_durva_* and 500 Gy of Y90-RE were 49.8% (95% CI, 46.8–53.0%), 51.4% (95% CI, 48.4–54.6%), and NA.

In arm C, the 1-, 3-, and 5-year DM rates from 0.08 of *δ_durva_* and 300 Gy of Y90-RE were 59.4% (95% CI, 56.3–62.4%), 99.4% (95% CI, 98.7–99.7%), and NA. The 1-, 3-, and 5-year DM rates from 0.12 of *δ_durva_* and 400 Gy of Y90-RE were 49.1% (95% CI, 46.1–52.3%), 85.2% (95% CI, 83.0–87.4%), and NA. The 1-, 3-, and 5-year DM rates from 0.16 of *δ_durva_* and 500 Gy of Y90-RE were 46.2% (95% CI, 43.2–49.4%), 62.9% (95% CI, 60.0–65.9%), and NA.

We subsequently applied the model to investigate the effect of varying intervals of days between the administration of two drugs on treatment outcomes. The difference in administration interval days from 30 to 300 days was evaluated. Additionally, the intensity of the drugs was assessed by dividing them into three categories: minimum (0.08 of *δ_durva_* and 300 Gy of Y90-RE), intermediate (0.12 of *δ_durva_* and 400 Gy of Y90-RE), and maximum (0.16 of *δ_durva_* and 500 Gy of Y90-RE). The model predicts substantial changes in endpoint time with different interval days. Longer interval days negatively affect the efficacy of both targeted and non-targeted tumors. For the targeted tumor, the median PFS from the intensity of minimum, intermediate, and maximum decreases up to 7.4~15.2 months, 13.6~29 months, and 8.24~45 months, respectively. For the non-targeted tumor, the cumulative DM rates at 3 years from the intensity of minimum, intermediate, and maximum increased up to 0.4~0.5%, 8.3~10.4%, and 24.1~26.1% (Supplementary Figures S5 and S6).

The log-rank test was used to assess the significant difference in the treatment interval days between the two drugs and the intensity of the drugs, with a significance level of *p* < 0.05. Supplementary Figures S7 and S8 show the simulation results.

For the targeted tumor from 0.08 of *δ_durva_* and 300 Gy of Y90-RE, there were no statistically significant differences between arms A and B or arms B and C when the number of administration interval days of two drugs was less than 30 days (*p* < 0.25). However, the median PFS from arm A was significantly longer than that of arms B and C when the number of administration interval days of the two drugs was more than 30 days, and these three groups were significantly associated with PPS from 60 days or more (*p* < 0.05). Of the 0.12 of *δ_durva_* and 400 Gy of Y90-RE, there were no statistically significant differences between arms A and B when the number of administration interval days for two drugs was less than 30 days (*p* < 0.22). All three groups had a substantial association with PFS from 60 days or longer (*p* < 0.05). For the 0.16 of *δ_durva_* and 500 Gy of Y90-RE, when the interval between drug administrations was less than 60 days, no statistically significant differences were observed between arms A and B (*p* < 0.57). There was a significant correlation with PFS between all three groups from 90 days or longer (*p* < 0.05), as shown in Supplementary Figure S7.

For the non-targeted tumor from 0.08 of *δ_durva_* and 300 Gy of Y90-RE, the cumulative DM rate from arm A was considerably higher than arms B and C when the administration interval days between the two drugs exceeded 30 days (*p* < 0.05). However, no statistically significant differences were observed between arms B and C when the number of administration interval days of the two drugs was below 60 days (*p* < 0.21). The three groups were significantly related to the cumulative DM rates for 90 days or longer (*p* < 0.05). Using 0.12 of *δ_durva_* and 400 Gy of Y90-RE, the cumulative DM rate was similar in arms A and B when the number of administration interval days for the two drugs was below 30 days (*p* < 0.06). The cumulative DM rate from arm A was significantly lower than that of arms B and C when the interval of days between the two drugs was 60 days or longer (*p* < 0.05). Concerning the 0.16 of *δ_durva_* and 500 Gy of Y90-RE, when the interval between drug administrations was less than 90 days, no statistically significant differences were observed between arms A and B (*p* < 0.06). There was a significant correlation with the cumulative DM rates between all three groups from 120 days or longer (*p* < 0.05), as demonstrated in Supplementary Figure S8.

## 4. Discussion

Clinical trials are necessary to evaluate new cancer treatments and support evidence-based clinical decision-making. Successful cancer treatment for many indications relies on combination therapies. Several studies have shown the efficacy and tolerability of Y90-RE and ICI combination therapy, but the sequencing of Y90-RE/ICIs has not been determined [29,30]. The establishment of an optimal and effective treatment schedule is a significant obstacle to the clinical application of combination treatment regimens, as sequencing, dose, and choice of ICIs are parameters that can affect efficacy. Mathematical models can be utilized in a wide variety of ways to reduce the need for costly, labor-intensive, and time-consuming clinical studies. Thus, we present a mathematical model combining Y90-RE with ICIs as a treatment for locally advanced HCC patients to investigate treatment efficacy according to sequence and dose. The ultimate purpose of this model is not to encompass all pertinent underlying biological mechanisms but rather to focus on the Y90-RE and ICI parameters that we can adjust (sequence and dose) to guide the design of clinical trials. In addition, we intentionally included no term for simulating the direct response of immune responses to radiation but only indirect responses through tumor load reduction. In the future, subsequent clinical data will provide insight into the necessity of such a term and enable its parameterization.

Durvalumab was used in this study due to the availability of monotherapy-fitting data [45]. However, this model can be modified and utilized in the future with other types of ICIs as single-agent therapies. We independently fit both single-approach ICIs [45] and Y90-RE [47,48] models based on virtual patient populations and simulate treatment outcomes with the currently ongoing ICI-Y90-RE combination trial protocol [29,30].

Our model exclusively takes into account the radiosensitivity parameters for tumors ($\alpha_{T}/\beta_{T}$) while administering radiation, without considering the liver parenchyma. Hence, this study focuses only on the absorbed dose by the tumor, suggesting that it is equivalent to segmentectomy rather than lobectomy.

The results of this study predict several behaviors that have been clinically observed. Supplementary Figure S4 revealed that the benefit of adding Y90-RE to ICIs is increasingly improved in both targeted and non-targeted tumors than in patients who have only received a single treatment, as shown by previously small retrospective clinical trials [20,24,25]. The benefits of adding Y90-RE to ICIs are progressively enhanced in patients where less disease is left untreated. This needs to be considered when evaluating the efficacy of Y90-RE and ICI combination treatment, as this factor can differ significantly between patients and clinical trials. Research has shown that irradiating a small tumor burden has little effect on efficacy [49]. Conversely, it has been suggested that radiation to a larger tumor could potentially enhance the effectiveness of treatment outcomes [50]. Furthermore, we found an optimal treatment sequence of combination strategies. The results show that concurrent administration has significantly improved median PFS and 3-year DM rates than sequential therapies for targeted and non-targeted tumors, as shown in Figure 3 and Figure 4. For targeted tumors, in sequential treatment, the median PFS from arm B was improved compared to arm C (Figure 3). These findings demonstrated that immune activation was caused following radiotherapy or locoregional therapy in the preclinical data [21,22,23]. In addition, our results suggest that administering Y90-RE could potentially elicit an immunogenic effect, considering its capability to deliver higher radiation doses to targeted tumors [21,22]. Preclinical data have demonstrated that a combination of Y90-RE and ICI treatment produces a more robust anti-tumor immune response [26,27,28,29,30]. Of non-target tumors, in sequential treatment, the 1-year DM rate of arm C was lower than arm B, whereas at the 3-year DM rate, arm B was lower than arm C (Figure 4). These data show that patients may have experienced lymphopenia, decreased lymphocyte proliferation capacity, and loss of inflammatory cytokine production capacity immediately after the treatment [46,51].

As mentioned in the above results, we found a treatment strategy by considering the injection interval days between the two drugs and the treatment sequence, as illustrated in Supplementary Figures S7 and S8. Simultaneous administration of Y90-RE and ICI demonstrates the best therapeutic effect. However, if simultaneous injection is not possible, prolonging the treatment interval between the two drugs in sequential treatment, injecting Y90-RE first improves treatment results. Furthermore, as the *δ_durva_* and Y90-RE increase, the point at which a statistically significant difference between arms A and B occurs in the number of interval days between two drug injections increases. Thus, to optimize the treatment outcomes, the number of interval days between the two drug injections should be reduced to a shortened duration as the two medications decrease.

This model currently has several limitations, as follows: (a) Our present model does not predict absolute results for individual patients. Although all hypothetical patient populations were generated based on the realistic distribution of model parameters reflecting trends in the experimental data, the model does not predict outcomes absolute outcomes for individual patients. Instead, we can evaluate the results of different Y90-RE and ICIs regimens for representative HCC populations that fit HCC population-averaged parameters to the model. (b) No normal tissue and systemic toxicity are considered for Y90-RE and ICIs. In this study, we assume all regimens have similar degrees of adverse effects. However, treatment-related toxicities, disease severity, the extent of liver function impairment at diagnosis, and patient performance status influence allocation to HCC patients, posing significant challenges in achieving favorable treatment outcomes. Therefore, future enhancements might involve including dedicated toxicity models to verify that maximizing anti-tumor efficacy does not lead to intolerable levels of harm to normal tissues. This would require a substantial quantity of patient toxicity data for various Y90-RE and ICI combination regimens. (c) The model includes immunosuppressive mechanisms of Y90-RE, but it does not take into account that it might boost the effects of Y90-RE demonstrated in preclinical studies. If these enhancing effects of Y90-RE are observed in patient studies, they can be accounted for by changing $\omega_{1}$ to depend on dosage size. (d) The tumor is currently depicted as a single compartment without addressing various spread patterns. Tumors can be added in multiple compartments of the lung, liver, kidney, and brain, with distinct antigen-stimulating and infiltrating lymphocyte dynamics as proposed by Walker et al. [43,52] and Serre et al. [53].

Despite these limitations, the major strengths of this study can assist in guiding the design of optimization treatment strategies in HCC patients and as a useful clinical tool in the emerging landscape of combination therapy. Additional clinical data for combination therapy will provide a foundation for continuous investigation and further iteration of the model to improve its accuracy and precision.

## 5. Conclusions

We developed a framework that integrates the effects of Y90-RE and ICIs and their possible synergy through tumor burden reduction and antigen release. The proposed mathematical model reproduces several relationships observed in clinical data and enables the optimization of combination strategies for HCC patients. Simulations of the model demonstrate that concurrent use of Y90-RE and ICIs yields greater benefits compared to their separate administration on consecutive interval days. In sequential treatment, if simultaneous injection is not possible, to optimize treatment results, the number of interval days between ICI and Y90-RE injections should be minimized as the intensity of ICIs and Y90-RE increases. Furthermore, injecting Y90-RE first improves treatment outcomes. This model has the potential to function as a simulation tool for improving the decision-making process regarding the optimal timing and dosage of Y90-RE with ICIs and maybe other types of anticancer therapies. Future research will expand this model to other clinical datasets and find a way to manage separation into different tumor compartments, radiation, and drug-induced adverse effects in the optimization process.

## Figures and Tables

**Table 1:** Summary of the model parameters.

Parameter	Function	Value	Ref.
a	Tumor growth	$0.01\;d^{-1}$	[37]
f	Lymphocyte decay rate	$0.033\;d^{-1}$	[38]
$\alpha_{T}/\beta_{T}$	Tumor—LQ cell death	10 Gy	[39]
r	Inactivated tumor cell decay rate	$0.14\;d^{-1}$	[40]
$\omega_{1}$	Tumor-directed lymphocyte efficiency	$0.119\;d^{-1}$	[33,34]
$\omega_{2}$	Tumor/inactivated tumor	$0.003\;d^{-1}$	
$\omega_{3}$	Tumor–lymphocyte recruitment constant	$0.009{\;d}^{-1}$	[33,34]
g	Geometric saturation constant	$7.330\times 10^{10}\;$	[33,34]
s	Lymphocyte regeneration	$1.470\times 10^{8}{\;d}^{-1}\;$	[41]
$\alpha_{T}$	Tumor—LQ cell death	Normally distributed (μ=0.148 σ=0.024)	[39]
$\alpha_{L}$	Lymphocytes—LQ cell death	$0.737{\;Gy}^{-1}$	[42,43]
*C_max_*	Maximum concentration	10 mg/kg	[44]
T(ω)12	Half-life of immune checkpoint inhibitor (durvalumab) in the body	21 days	[44]
*δ_durva_*	Effectiveness of immune checkpoint Inhibitor (durvalumab)	Normally distributed (μ = 0.12 σ = 0.04)	[45]
T(Y90RE)12	Half-life of yttrium 90 in the body	2.6 days	[36], added in this study
*q*	Effectiveness of yttrium 90	Normally distributed (μ = 0.12 σ = 0.04)	This study, added in this study
*k*	Constant to produce the dose rate in desired units	(0.9267 Mevdis)·(1.6022×10−13JMev)	[36], added in this study
*<E>*	Average energy emitted per nuclear transition	(GykgJ)·(109dissGBq)·(86400sday)	[36], added in this study

## Data Availability

Data can be obtained from the corresponding author upon reasonable request.

## Supplementary Materials

The following supporting information can be downloaded at: https://www.mdpi.com/article/10.3390/bioengineering11020106/s1, Figure S1: Modeling the synergistic impact of yttrium 90 radioembolization and immune checkpoint inhibitors on hepatocellular carcinoma.
