# Supplementary material for: Modeling the Synergistic Impact of Yttrium 90 Radioembolization and Immune Checkpoint Inhibitors on Hepatocellular Carcinoma
Source: Bioengineering (Basel). 2024 Jan 23;11(2):106. doi: 10.3390/bioengineering11020106 (PMC10886259; doi:10.3390/bioengineering11020106)
Supplement: Supplementary file 1 [file bioengineering-11-00106-s001.zip › bioengineering-2813057-supplementary.pdf]

Supplementary materials: Modeling the synergistic impact of Yttrium-90 radioembolization and immune checkpoint inhibitors on hepatocellular carcinoma

Model Equations.

$$\frac{dT_I}{dt} = \overbrace{aT_I}^{Tumor\ growth} \quad \overbrace{-\omega_1' \frac{T_I}{g+T_I+M}\ \mathrm{L}}^{immune-induced\ death} \quad \overbrace{-\delta^*(t_R)\left(1-e^{-\alpha_T D_T-\beta_T D^2{}_T}\right) T_I}^{radiation\ kill\ from\ Yttrium-90} \qquad \text{(a)}$$

$$\frac{dT_{NI}}{dt} = \overbrace{aT_{NI}}^{Tumor\ growth} \quad \overbrace{-\omega_1' \frac{T_{NI}}{g+T_I+T_{NI}}\ L}^{immune-induced\ death} \qquad \text{(b)}$$

$$\frac{dI}{dt} = \overbrace{-\delta^*(t_R)\left(1-e^{-\alpha_T D_T-\beta_T D^2{}_T}\right) T_I}^{radiation\ kill\ from\ Yttrium-90} \quad \overbrace{-\mathrm{rI}}^{decay} \qquad \text{(c)}$$

$$\frac{dL}{dt} = \overbrace{\omega_2 \frac{T_I+T_{NI}}{g+T_I+T_{NI}}\ L\ +\ \omega_3 \frac{I}{g+I}\ L}^{recruitment} \quad \overbrace{+s}^{supply} \quad \overbrace{-fL}^{decay} \overbrace{-\ \sum_i \delta^*(t_R)(1-e^{-\alpha_L D_{L_i}})L_i}^{radiation\ kill\ from\ Yttrium-90} \qquad \text{(d)}$$

$$\omega_1'(t) = \qquad \overbrace{\omega_1(1+\delta_{durva}\cdot C_{max} e^{\frac{-\ln(2)t}{t_{(\omega)1/2}}})}^{immune\ checkpoint\ inhibitors-\ induced\ death} \qquad \text{(e)}$$

(a) The compartment of targeted tumor cells is a function of the intrinsic tumor growth rate (*a)* reduced by its cell kill induced by lymphocytes (ω_1') and radioactivity. We use a Monod Form ($T\ /\ (g+T)$), which is also call Michaelis-Menten term) with a half-saturation constant g. This form considers the saturation effects because, as described by Kirshner and Panetta [1], the reduced fraction of the increasing tumor volume being in contact with circulating lymphocytes.

(b) The non-targeted population (T_{NI}) are considered to have the same growth as targeted tumor cell (T_I).

(c) the number of circulating lymphocytes (*L*) is a function of lymphocyte recruitment by tumor growth(ω_2) and inactivated tumor cells(ω_3,), regeneration(*s*), simultaneously decay exponentially with time (*f*), and immune cell kill of radiation.

(d) inactivated tumor cells (*I*) that are irradiated, which stimulate immune response, decay with a constant rate (*r*).

(e) the tumor cell kill rate (ω_1'), which ascertains the effector-lymphocyte coupling and thereby simulates the increase in cell kill by effector lymphocytes induced by immune checkpoint inhibition, is a time-dependent function of the intrinsic anti-tumor immune response (ω_1), plus a patient specific immune checkpoint inhibitor response (δ_{durva}), the maximum drug concentration (C_{max}) and the half-life of the immune checkpoint inhibitor ($t_{1/2}$).

1. Initial distributions of the parameters in a virtual patient cohort.

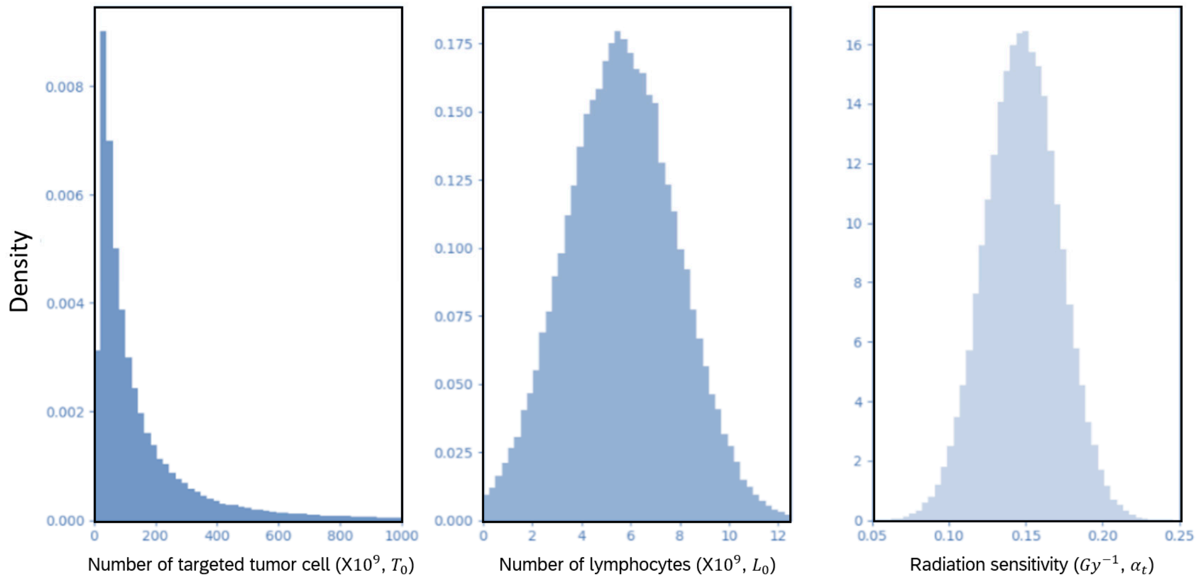

Supplementary Figure S1. The initial distributions of tumor, lymphocytes, and radiation sensitivity from which we take a random sample for each iteration of a single individual patient.

2. Response rate the virtual population of 10,000 patients

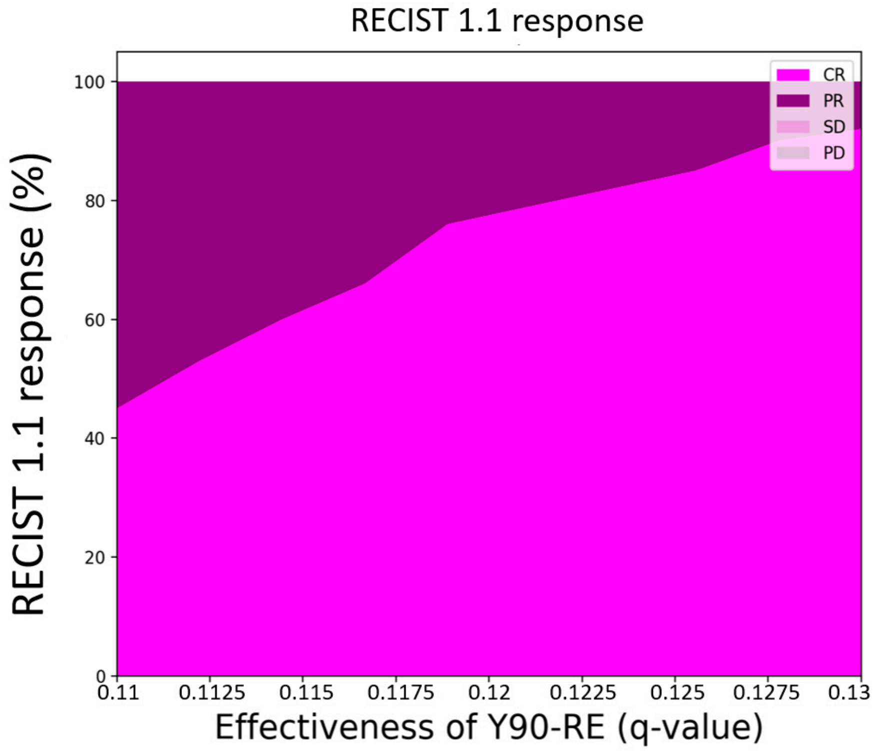

Supplementary Figure S2. For 10,000 virtual patients, the impact of yttrium 90 on RECIST 1.1 response. The green, blue, orange, and red lines indicate complete response (CR), partial response (PR), stable disease (SD), and progressive disease (PD), respectively. Abbreviations: RECIST = The response evaluation criteria in solid tumor, CR = complete response, PR = partial response, SD = stable disease, PD = progressive disease

3. Distribution of effectiveness of yttrium-90 (q)

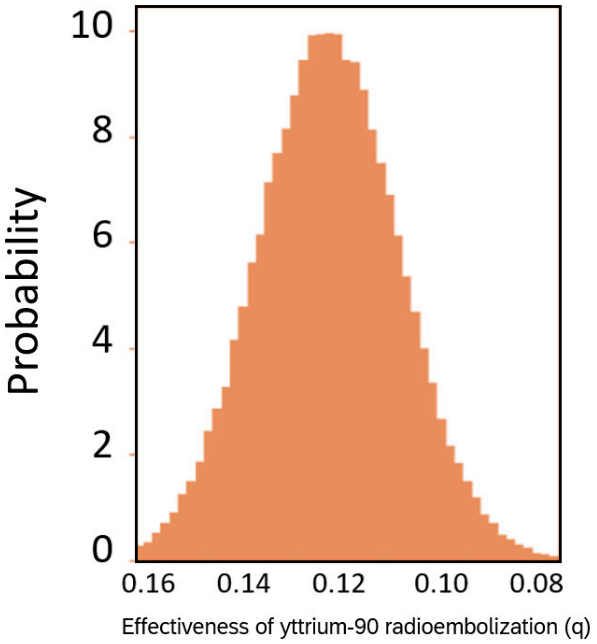

Supplementary Figure S3. A figure was used to find RECIST 1.1 responses and suitable-for-reference value distribution. The distribution of yttrium-90 suitable for the values for CR (79%), PR (20%), SD (0%), and PD (0%), based on the RECIST 1.1 response to the constant q. Abbreviations: PR = partial response; SD = stable disease; PD = progressive disease; RECIST = The response evaluation criteria in solid tumors

4. Model prediction (Combination therapy vs monotherapy)

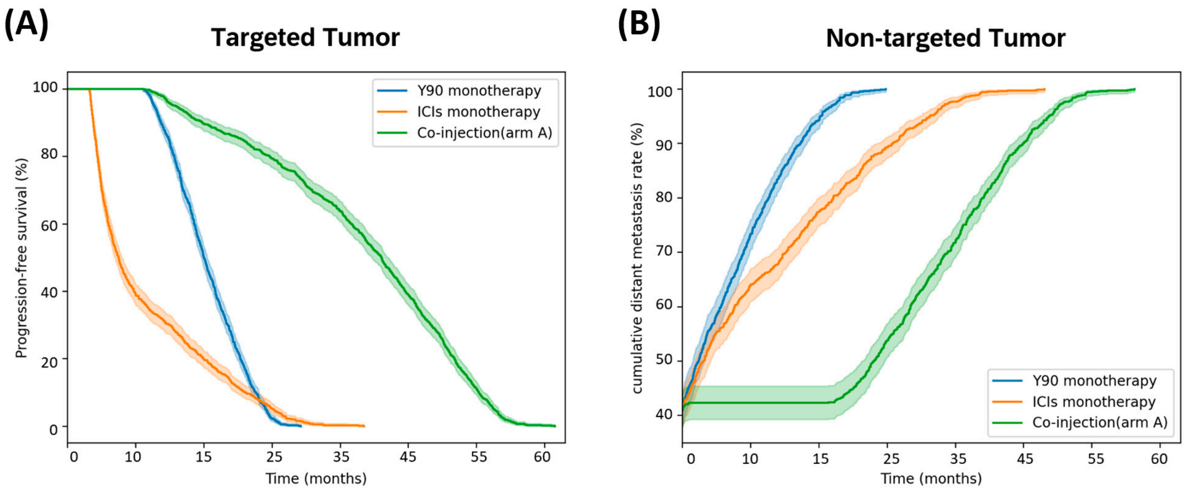

Supplementary Figure S4. (A) The progression-free survival (PFS) for Co-injection (Green), Y90-RE monotherapy (Blue), and ICIs monotherapy (Orange). The median PFS from arm A, B, and C was 39.3 months (95% CI, 37.2-39.2 mo), 16.2 months (95% CI, 16.3-17 mo), and 6.2 months (95% CI, 5.8-6.6 mo). The 1-, 3-, and 5-years PFS rates from arm A were 96.4% (95% CI, 94.8-97.3%), 54.9% (95% CI, 51.8-58%), and NA. The 1-, 3-, and 5-years PFS rates from arm B were 86.1% (95% CI, 83.7-88.0%), NA, and NA. The 1-, 3-, and 5-years PFS rates from arm C were 30.4% (95% CI, 27.5-33.2%), NA, and NA. (B) The cumulative distant metastasis (DM) for Co-injection (Green), Y90-RE monotherapy (Blue), and ICIs monotherapy (Orange). The 1-, 3-, and 5- years DM rates from arm A was 42.3% (95% CI, 39.4-45.5%), 79.7% (95% CI, 77.2-82.2%), and not available (NA). The 1-years DM rates from arm B was 85% (95% CI, 82.8-87.2%), and the 3-, and 5-years DM rates from B were NA. The 1-, 3-, and 5- years DM rates from arm C was 68.9% (95% CI, 66.5-72.2%), 99.9% (95% CI, 98.7%-NA), and NA. Abbreviation: Y90-RE =Yttrium 90 radioembolization, ICIs =immune checkpoint inhibitors.

5. The progression-free survival (PFS) heatmap for timing of administration and interval days (Targeted tumor)

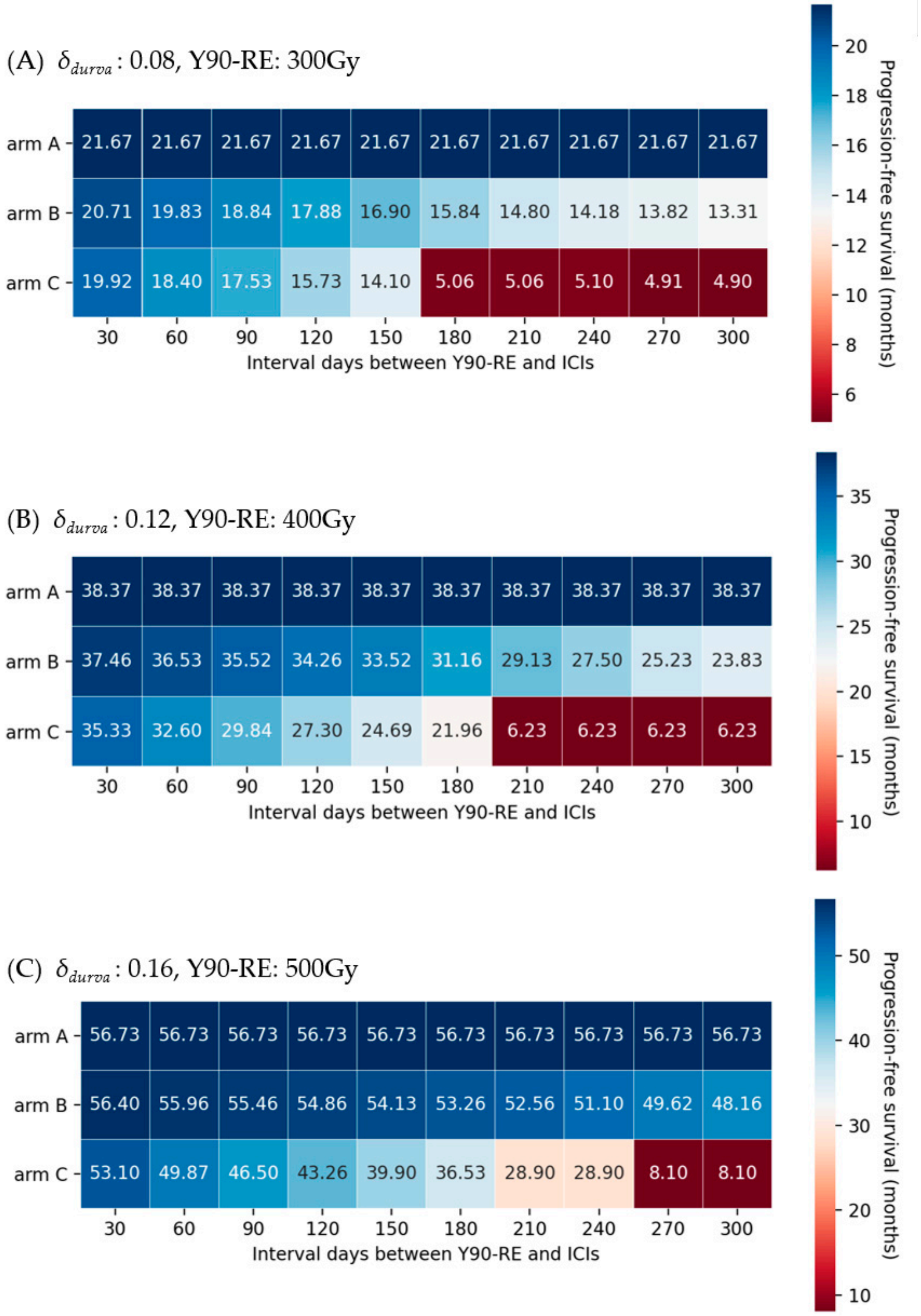

Supplementary Figure S5. The progression-free survival (PFS) heat maps for timing of administration and interval days for three arm (A, B, and C) for the targeted tumor. The color columns indicate the median PFS months. The interval days of administration between Y90-RE and ICIs evaluated from 30 to 300 days. Abbreviations : Y90-RE = Yttrium 90 radioembolization, ICIs = Immune checkpoint inhibitors.

6. The cumulative distant metastasis (DM) heatmap for timing of administration and interval days (Non-targeted tumor)

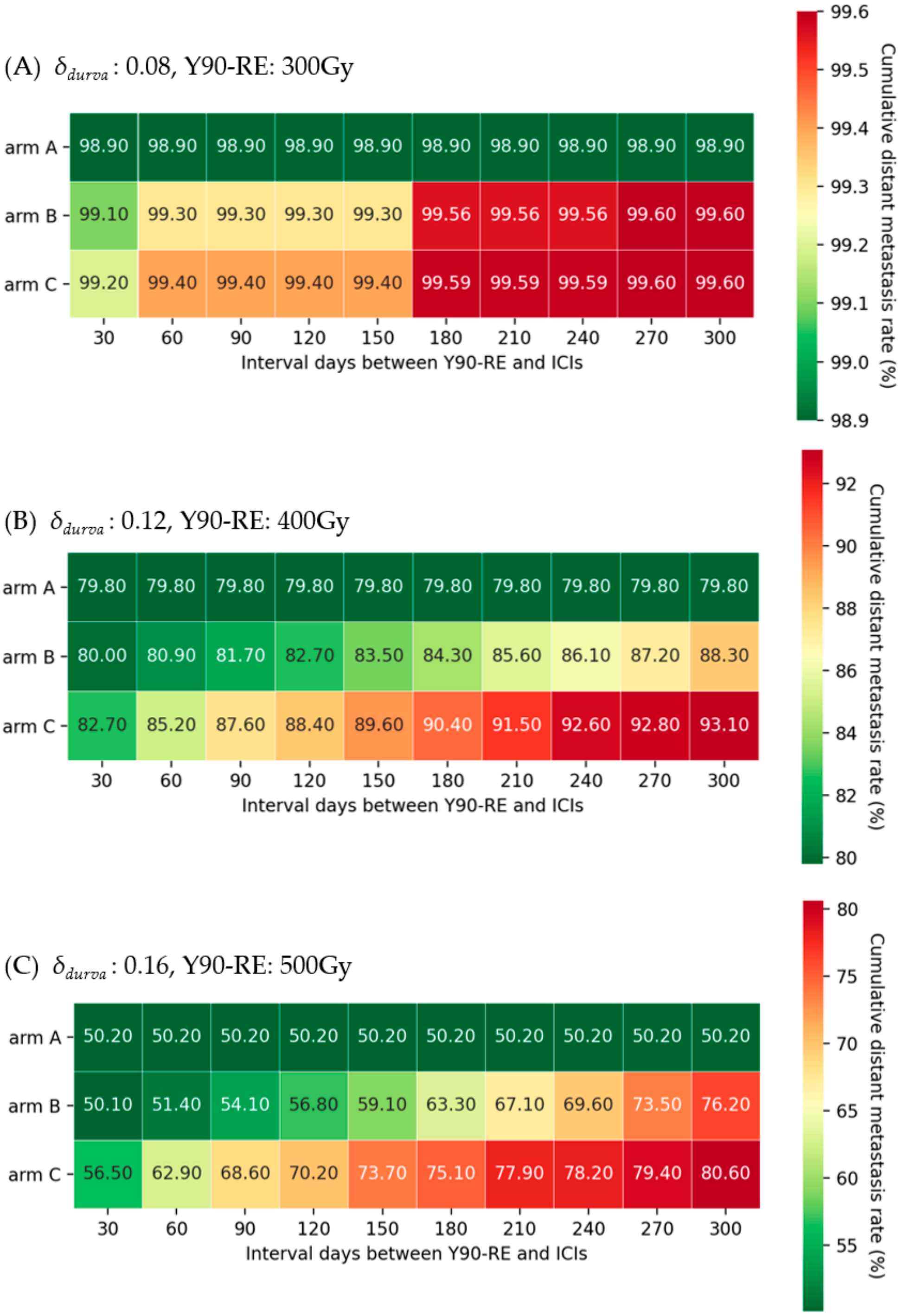

Supplementary Figure S6. The cumulative distant metastasis (DM) at 3-years heat maps for timing of administration and interval days for three arm (A, B, and C) for the non-targeted tumor. The color columns indicate the median DM months. The interval days of administration between Y90-RE and ICIs evaluated from 30 to 300 days. Abbreviations : Y90-RE = Yttrium 90 radioembolization, ICIs = Immune checkpoint inhibitors.

7. The p-value heatmap for timing of administration and interval days (Targeted tumor)

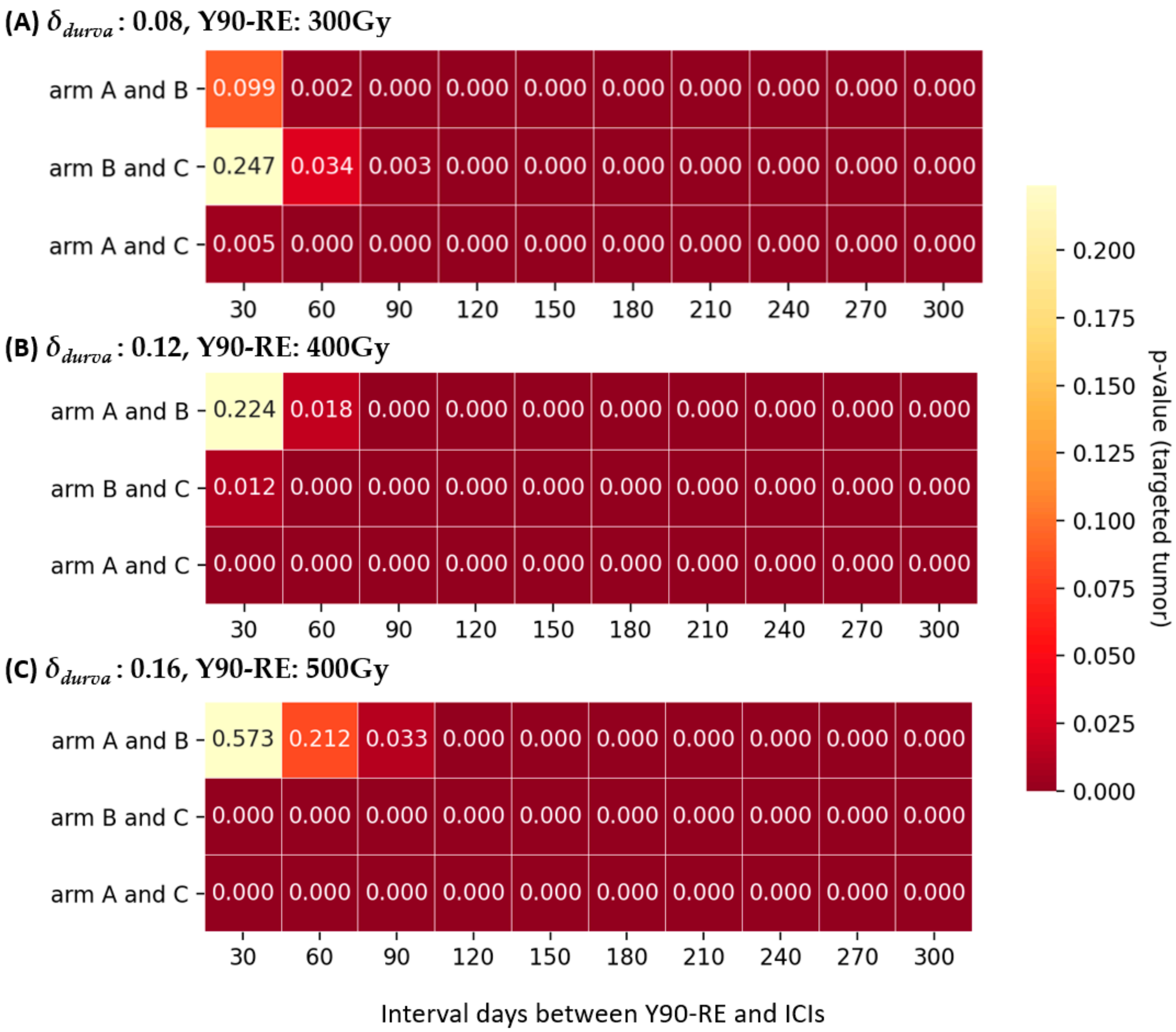

Supplementary Figure S7. The p-value heat maps for timing of administration and interval days for three arm (A, B, and C) for the targeted tumor using Log-rank test. Arm A is co-injection, arm B is Y90-RE first injection, and arm C is ICIs first injection. The color columns indicate the p-value. The interval days of administration between Y90-RE and ICIs evaluated from 30 to 300 days. Abbreviations : Y90-RE = Yttrium 90 radioembolization, ICIs = Immune checkpoint inhibitors.

8. The p-value heatmap for timing of administration and interval days (Non-targeted tumor)

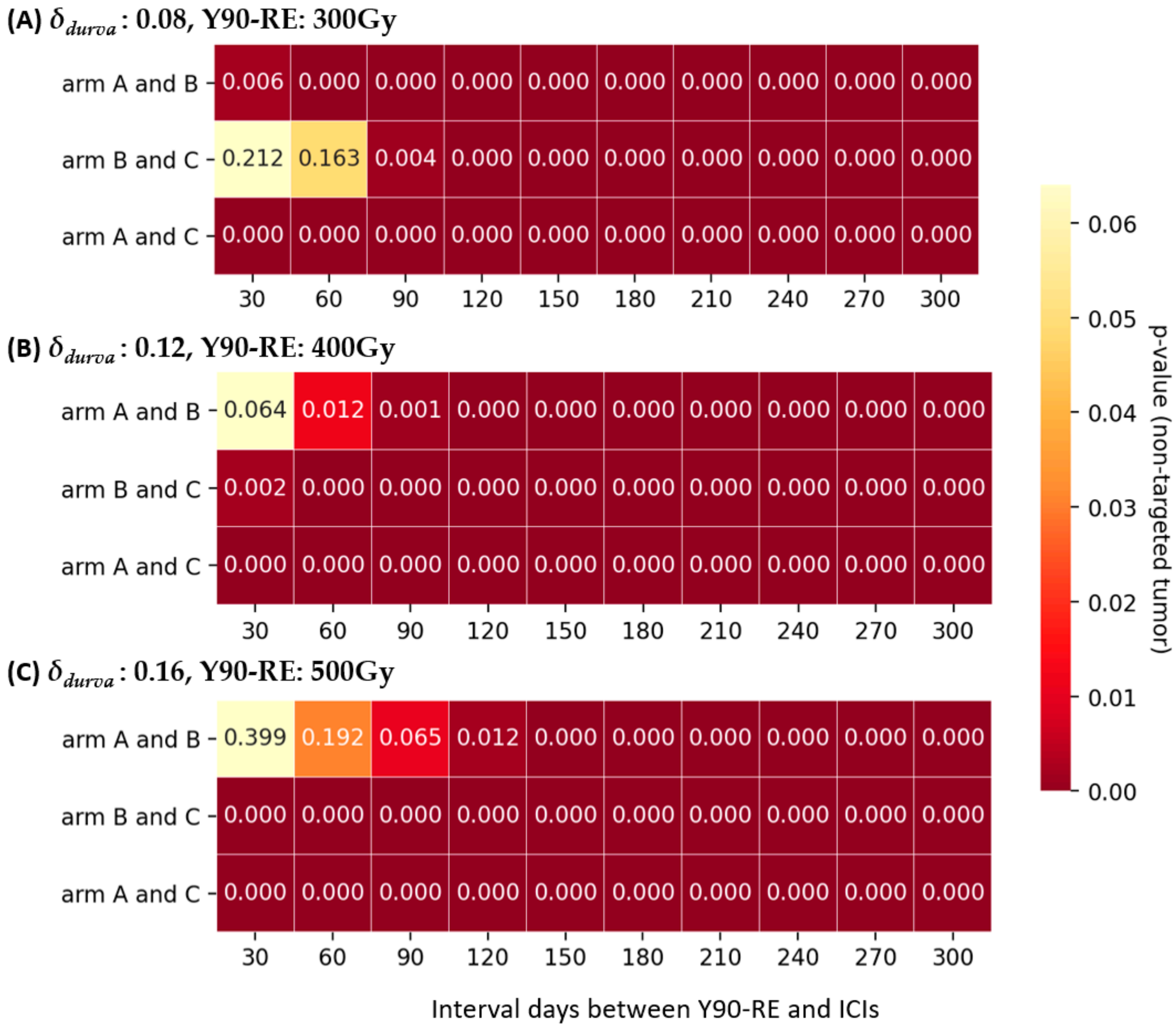

Supplementary Figure S8. The p-value heat maps for timing of administration and interval days for three arm (A, B, and C) for the non-targeted tumor using Log-rank test. Arm A is co-injection, arm B is Y90-RE first injection, and arm C is ICIs first injection. The color columns indicate the p-value. The interval days of administration between Y90-RE and ICIs evaluated from 30 to 300 days. Abbreviations : Y90-RE = Yttrium 90 radioembolization, ICIs = Immune checkpoint inhibitors.

References

1. Sotolongo-Grau, O., Rodríguez-Pérez, D., Santos-Miranda, J. A., Sotolongo-Costa, O., & Antoranz, J. C. (2009). Immune system-tumour efficiency ratio as a new oncological index for radiotherapy treatment optimization. *Mathematical Medicine and Biology*, *26*(4), 297–307. https://doi.org/10.1093/imammb/dqp005
